# Antibiotic profiling of multidrug resistant pathogens in one-day-old chicks imported from Belgium to benin

**DOI:** 10.1186/s12917-023-03570-y

**Published:** 2023-01-21

**Authors:** Philibert Dougnon, Victorien Dougnon, Boris Legba, Kafayath Fabiyi, Arnaud Soha, Hornel Koudokpon, Kevin Sintondji, Esther Deguenon, Gildas Hounmanou, Carlos Quenum, Taératou Aminou, Richard Lokossou, Innocent Togla, Cyrille Boko, Bruno Djossa, Françoise Assogba-komlan, Lamine Baba-moussa

**Affiliations:** 1 Research Unit in Applied Microbiology and Pharmacology of Natural Substances, Research Laboratory in Applied Biology, Polytechnic School of Abomey-Calavi, University of Abomey-Calavi, Abomey-Calavi, Benin; 2 Global Veterinary Agency, Cotonou, Benin; 3 Ministry of Agriculture, Livestock and Fisheries, Cotonou, Benin; 4 Communicable Diseases Research Unit, Research Laboratory in Applied Biology, Polytechnic School of Abomey-Calavi, University of Abomey-Calavi, Abomey-Calavi, Benin; 5 Forestry and Bioresource Conservation Research Unit, School of Tropical Forestry, National University of Agriculture, Abomey-Calavi, Benin; 6 Laboratory of Biology and Molecular Typing in Microbiology, Faculty of Sciences and Techniques, University of Abomey-Calavi, Abomey-Calavi, Benin

**Keywords:** Imported one-day-old chicks, Resistance gene, Pathogenic bacteria, Benin

## Abstract

**Background:**

Little data exist on the presence of resistant pathogens in day-old chicks imported into Benin. The occurrence of pathogenic bacteria was assessed in 180 one-day-old chicks imported from Belgium and received at the Cardinal Bernardin Gantin International Airport in Cotonou (Benin). The samples included swabbing the blisters of 180 chicks, followed by 18 pools of 10 swabs for bacterial isolation. Classic bacteriological methods based on Gram staining, culture on specific media and biochemical characterization were used. Antibacterial susceptibility screening to antibiotics was conducted using the Kirby–Bauer disc diffusion method, and the results were interpreted according to guidelines from the European Committee on Antimicrobial Susceptibility Testing (EUCAST). DNA extraction was performed by the heat treatment method. Resistance genes were screened by real-time PCR.

**Results:**

We isolated 32 bacteria, including *Escherichia coli* (50%), *Enterococcus spp.* (28%), and coagulase-negative staphylococci (10%). The isolates were investigated for antibiotic resistance against antibiotics using the disk diffusion method and showed that in the *Escherichia col*i strains isolated, the highest rate of resistance was obtained against ciprofloxacin (81%), followed by trimethoprim + sulfamethoxazole (62%). *Enterobacter cloacae* was sensitive to all the antibiotics tested. *Pseudomonas spp.* resistant to amoxicillin and trimethoprim + sulfamethoxazole was noted. The *SulII* gene was found in all cloacal samples, while the *SulI* and *bla*_*TEM*_ genes were present at 44.44% and 16.67%, respectively.

**Conclusion:**

This study confirms that imported day-old chicks can be a potential source of dissemination of resistant bacteria in poultry production. A system for immediate detection of resistant bacteria in chicks upon arrival in the country is thus needed.

## Background

Poultry is one of the most widespread food industries in the world, and chicken is the most commonly raised species, with over 90 billion tons of chicken meat produced annually [1]. In both developed and less developed countries, chicken meat is among the most meaty of all meat products [2]. According to the Global Livestock Counts report, there are approximately 19 billion chickens in the world [3]. In 2019, the consumption of chicken meat in the United States was 16,700 tons; in Europe, it was 11,636 tons; and in India, it was 4347 tons [4]. In Africa, chicken is the most consumed meat, with 5.75 kg per capita per year between 2018 and 2020, ahead of beef, sheep and pork [5]. The main reasons for this interest in chicken meat are the relatively low production costs and the lack of cultural and religious restrictions on its consumption [1]. However, the absence of trans fats makes them a healthier option, as they are associated with cardiovascular diseases, while other meats, such as beef, have a large amount [6].

There is considerable scientific evidence that poultry can be affected by the growth of pathogenic bacteria, which can be a source of contamination of chicken meat. This applies to imported day-old chicks in which pathogens, some of which are resistant to antibiotics, have been detected [7, 8]. However, if the necessary preventive measures are not taken into account during the marketing, processing and production of chicken, eggs and chicken meat may be contaminated with infectious agents that could be pathogenic to humans [9].

*Campylobacter* and *Salmonella* are the most common pathogenic microbes that account for more than 90% of bacterial-associated food poisoning cases and are considered responsible for food safety risks worldwide [9]. Apart from these bacterial species, enterobacteria and some gram-positive bacteria are also responsible for food poisoning. *Escherichia coli* and *Salmonella* are the predominant bacteria found in the intestines of animals and humans. These microbes serve as indicators of fecal contamination of food and water due to untreated sewage discharges into natural waterways [3]. Chickens contaminated with *E. coli* are evidence of poor hygiene practices in slaughterhouses and commercial areas [3].

To address the problem of contamination, the poultry industry started using antibiotics to improve meat production by using fortified feeds for disease prevention [10]. The global livestock sector is responsible for 70% of antibiotic use [11, 12], while LMICs come under scrutiny due to high levels of poverty, inadequate healthcare facilities, poor sanitation and water treatment infrastructures, and weak regulatory and monitoring capacities [13]. A wide variety of antimicrobials are used to raise poultry in most countries, mainly orally, to prevent and treat disease and to improve growth and productivity [14]. Moreover, poor farmer education and low literacy levels entail a limited understanding of how antibiotic therapies function and are thus a lack of awareness of the risks of misuse [15].

Several studies have looked for antibiotic-resistant microorganisms considered critical to human health in the gut microbiota of day-old chicks [16–18]. Furthermore, there is clear scientific evidence that imported day-old chicks can carry antibiotic resistance genes. The work of Coppola et al. [8] reported that day-old chicks imported from Brazil are sources of the hidden spread of pathogens resistant to WHO priority antibiotics. This study shows that bacteria isolated from day-old chicks harbor *mcr-9, rmtG* and β-lactam resistance genes, which is a critical issue from a One Health perspective. Multidrug-resistant bacteria carried silently by day-old chicks could be a significant source of antimicrobial resistance problems and infection of humans through food. If imported day-old chicks carry antibiotic resistance genes, these strains can be transmitted to other chicks or animals on local farms. Resistant strains can therefore spread in the environment or be transmitted to humans, which can cause health problems. Thus, it is likely that highly antimicrobial-resistant bacteria are imported into Benin in day-old chicks and eggs and are spreading throughout the production chain, prompting hard-to-treat infections, subsequent excessive use of antimicrobials, and contamination of consumed poultry eggs and meat [19].

In Benin, there are few data on the presence of resistant pathogenic bacteria in imported day-old chicks. However, imported day-old chicks are known to come mainly from Europe: Belgium, France, Germany, Brazil, and the Netherlands. In Africa, it concerns South Africa and Nigeria [3]. The present study was initiated to provide data on the microbiological quality of day-old chicks in Benin. The objective of this study was to characterize antibiotic-resistant bacteria in day-old chicks imported into Benin. The study focuses on day-old chicks imported from Belgium to Benin.

## Results

### Collection and identification

**Fig. 1:**
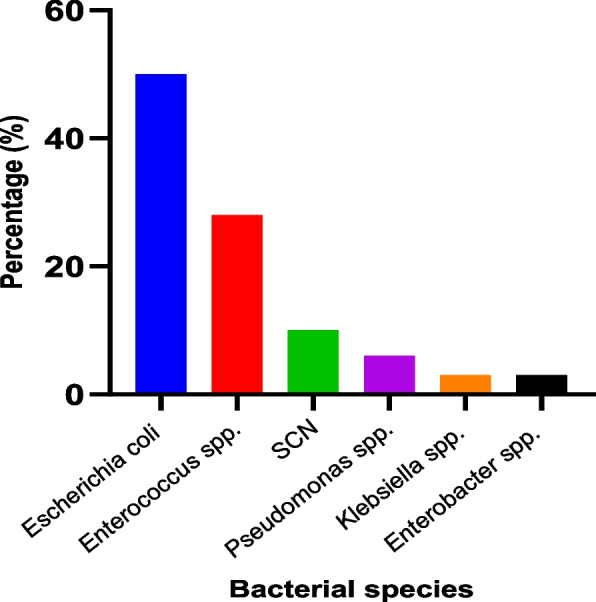
Frequency of bacterial species isolated

Twenty-four hours after the samples were cultured, bacterial growth was noted on several Petri dishes. Gram staining showed the presence of gram-negative bacilli in all sample pools, while only eleven sample pools showed the presence of gram-positive cocci. A total of 32 bacteria were isolated from the 18 sample pools. The most frequently identified bacteria were *Escherichia coli* (50%), *Enterococcus spp.* (28%), and coagulase-negative Staphylococci (10%). Other strains isolated include *Pseudomonas spp, Klebsiella spp* and *Enterobacter spp.* These results are presented in Fig. 1.

### Antibiotic resistance profile of isolated strains

#### The resistance profile of the isolates to antibiotics was determined

**Fig. 2:**
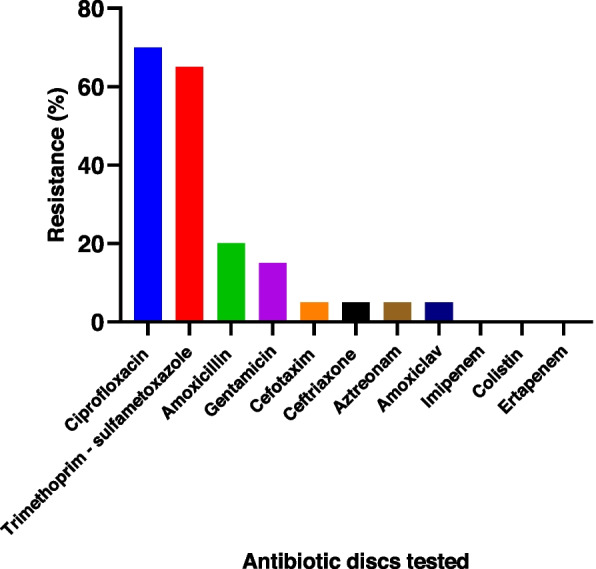
Resistance profile of isolated Gram-negative bacilli to antibiotics

**Fig. 3:**
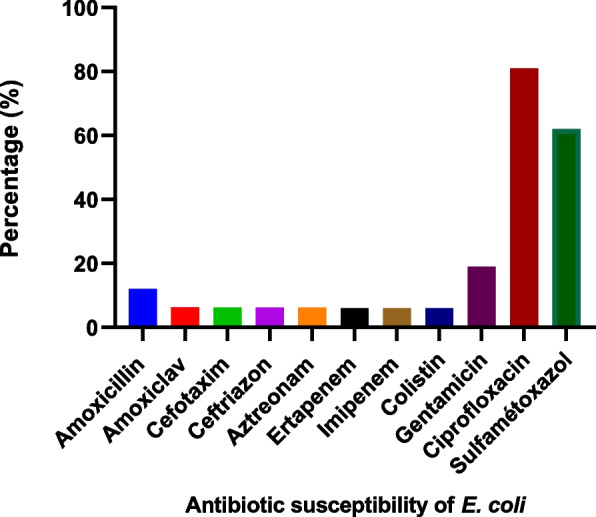
Antibiotic susceptibility of *E. coli*

gram-negative bacilli: No resistance of gram-negative bacilli was noted to carbapenems or to colistin (Fig. 2). Of the 16 *Escherichia coli* isolates, the highest rate of resistance was obtained against ciprofloxacin (81%), followed by trimethoprim + sulfamethoxazole (62%) (Fig. 3). All isolates of *Klebsiella spp*. were resistant to ciprofloxacin, trimethoprim + sulfamethoxazole and amoxicillin. On the other hand, *Enterobacter cloacae* was sensitive to all the antibiotics tested, except trimethoprim + sulfamethoxazole (62%). However, one strain of *Pseudomonas spp.* was resistant to amoxicillin and trimethoprim + sulfamethoxazole.

### Coagulase-negative *Staphylococci*

The observed resistance concerns only gentamicin (33%) and tobramycin (33%).

#### *Enterococcusspp*

The resistances observed concern only oxacillin (33%) and ceftriaxone (100%).

#### Resistance gene detection

Concerning the detection of resistance genes, the SulII gene was found in all cloacal samples, while the SulI and *bla*_*TEM*_ genes were present at 44.44% and 16.67%, respectively. Carbapenem resistance genes were not detected in any sample. The same is true for the SHV, CTX-M and mcr-1 genes.

## Discussion

Due to insufficient domestic production, Benin imports a significant quantity of day-old chicks. To illustrate, from 2020 to 2021, Benin legally imported 271,380-day-old chicks through the Cotonou International Airport. No studies have been carried out on the screening of pathogens present in these chicks or their sensitivity to antibiotics. However, there is ample scientific evidence that the importation of day-old chicks can contribute to the introduction of resistant strains of bacteria into chicken farms [20, 21].

The aim of this study was to characterize antibiotic-resistant bacteria in day-old chicks imported into Benin. We screened 180-day-old chicks imported from Belgium to Benin for the presence of antibiotic-resistant pathogens and resistance genes.

In sampling, we opted to pool samples because they are more effective in predicting contamination than isolate pathogen individual samples [22]. The cloacal samples collected from the chicks were positive, allowing us to isolate bacilli such as *Escherichia coli, Klebsiella spp., Enterobacter spp., Pseudomonas spp.*, and cocci such as *Enterococcus faecalis* and coagulase-negative staphylococci. The number of isolated bacterial species was very low, indicating a low level of contamination. For the identification process, the API Gallery was used to confirm the primary identification tests performed with the Leminor Gallery. The number of isolates reported corresponds to those whose species could be identified by the test with the Gallery API 20.

Of the strains isolated, *Escherichia coli* and *Enterococcus spp.* were the most common, accounting for 50% and 28%, respectively. It is known that the gastrointestinal tract of poultry contains a relatively large and diverse bacterial community characterized by ecological niche specificity and synergy between the bacterial species in the community. Although the age of the chickens influences the characteristics and composition of this community, it has been shown that day-old chicks, after hatching, already have a microbial community in their gastrointestinal tract [23]. For example, the genera *Enterococcus* and *Staphylococcus* are found in the *small intestine and caeca. Escherichia* is found in the caeca and large intestine. *Enterobacter* is found in the gizzard [23]. Thus, at first sight, isolating these bacteria in the feces of chicks does not seem surprising, but there is evidence that imbalances in the synergy between the bacteria of the microbiota can lead to a pathological transition such that the commensal bacteria can become pathogenic and cause an infection [24]. For example, *E. coli* is one of the most common bacterial pathogens in poultry worldwide and is responsible for colibacillosis, which usually presents as a localized or systemic infection in poultry [25]. It is considered a major pathogen of global importance in commercially produced poultry [26]. It is therefore not surprising that the predominance of *Escherichia coli* in poultry was noted in several studies [16–18].

Isolation of *Klebsiella* spp. and *Pseudomonas aeruginosa* from the feces of the day-old chicks showed that bacterial contamination of the chicks had occurred. There are several hypotheses that could be the basis for the contamination at birth of day-old chicks. The infection can be transmitted either through the egg shell at the time of hatching, i.e., by horizontal or vertical transmission. Vertical transmission occurs through systemic infection of the ovaries or during sexual intercourse with a contaminated cloaca, affecting the vagina and the lower regions of the oviduct [27]. During vertical transmission, the yolk, albumen and membrane come into direct contact with contaminants due to bacterial infection of reproductive organs such as oviduct tissue and ovaries. As a result, eggs are contaminated before the shell is formed [4]. However, contamination of day-old chicks could occur through feed, hands, insects, and water. Contamination of day-old chicks can also occur through interaction with dust, feces and soil during transport and from caging equipment.

Among gram-positive cocci, *Enterococcus spp*. were the most identified. *E. faecalis* is considered an opportunistic pathogen. In poultry, it causes depression growth, pulmonary hypertension syndrome, and amyloid arthropathy, in addition to first week mortality [28].

In this study, little antibiotic resistance was observed, but the data obtained are quite worrisome for day-old chicks. Antibiotic susceptibility testing showed very low levels of resistance to the antibiotics tested. No resistance of gram-negative bacilli to carbapenems or colistin was noted. No resistance was noted to vancomycin and teicoplanin for coagulase-negative *Staphylococcus* and *enterococcus* strains. *Escherichia coli* isolates, the highest rate of resistance was obtained against ciprofloxacin (81%), followed by trimethoprim + sulfamethoxazole (62%). *Klebsiella spp.* strain is also resistant to ciprofloxacin and trimethoprim + sulfamethoxazole. This result confirms the observations made by Coppola et al., who observed the highest resistance of *E. coli* and *Klebsiella* to ciprofloxacin in day-old chicks imported from Brazil to Uruguay [20]. Resistance to ciprofloxacin, trimethoprim + sulfamethoxazole and gentamycin was previously noted in the work of Kilonzo-Nthenge et al. [29].

High resistance of gram-negative bacilli to ciprofloxacin, trimethoprim + sulfamethoxazole and gentamicin is a concern. This may be due to the unnecessary and inappropriate overuse of antimicrobial agents as feed additives or prophylactic treatments in chickens, as also reported by par Azad et al. [30]. Several data confirm the prophylactic use of antibiotics in chicken farms in Belgium [31]. Furthermore, these antibiotics are commonly used in humans. This situation could considerably increase the level and spread of multiresistant bacteria to antibiotics, which may cause human health management of infections due to resistant bacteria.

Coagulase-negative *Staphylococci were* resistant to gentamicin (33%) and tobramycin (33%), while *Enterococcus spp*. were resistant to oxacillin (33%) and ceftriaxone (100%). The use of antibiotics in chickens has certainly greatly improved their growth and health by strengthening their immune system [10]. However, the use of antibiotics can cause an allergic reaction and an imbalance of the gut microbiota and, in some cases, lead to the development of antibiotic resistance [32]. Noted resistance to antibiotics such as gentamicin, SXT, and ciprofloxacin can be explained by their uncontrolled use in treatment or as growth promoters or prophylactics in poultry [25, 33].

To understand the observed phenotypic resistances, the antibiotic resistance genes were searched by PCR. The *SulII* gene was found in all cloacal samples, while the *SulI* and *bla*_TEM_ genes were present at 44.44% and 16.67%, respectively. The results of sulfonamide resistance gene detection are in line with the phenotypic resistance profile observed. Sulfonamides are commonly used in animal health, but the persistence of this resistance can be due to the absence of selection pressure [34]. Sul genes are usually associated with mobile genetic elements such as plasmids; thus, the presence of *sul* genes in the studied isolates may indicate the presence of other resistance genes [35]. The presence of blaTEM genes is indicative of the ability of carrier strains to produce extended-spectrum β-lactamase (ESBL). This confirms previous work showing that the gastrointestinal tract of animals is seen as “an important reservoir for bacteria that produce β-lactamases and as a potential source for human pathogens to take up these resistance genes” [25]. The presence of the BlaTEM gene could explain the resistance of *E. coli* and *Klebsiella* to amoxicillin. Coppola et al. [20] reported the presence of BlaTEM-1b in *E. coli* isolated from day-old chicks imported from Brazil to Uruguay. The presence of antibiotic resistance genes reinforces the public health problem posed by the detection of pathogenic bacteria in imported day-old chicks. The possibility of transmission of this resistance to humans amplifies the issue and requires in-depth reflection for better surveillance of antimicrobial resistance in Benin.

### Limitations

We did not provide any information on the Belgian farms from which the imported chicks come: their health status, the history of the use of antibacterial agents in these farms, and the possible injection of certain antibacterial agents (such as gentamicin) into the embryonated eggs in the Belgian hatchery. When chicks are imported, such information is unfortunately not collected in Benin.

## Conclusion

The results presented in this study reveal the high prevalence of resistant pathogenic bacteria, including *E. coli*, in day-old chicks imported from Belgium to Benin. It is possible that these pathogens contaminate local farms and that they are transferred into the food chain in humans, with the possibility of causing health problems. It is necessary to set up a system for detecting antibiotic resistance in imported day-old chicks.

## Methods

### Sampling

Day-old chicks from Belgium received at the Cardinal Bernardin Gantin International Airport in Cotonou (Benin) were collected. The 180 chicks were divided into two batches of 90 chicks each, depending on whether they were of the Lohman or Novogen strains. Each batch of chicks was subdivided into 9 batches of 10 chicks, giving a total of 18 batches. The cloacae of the ten chicks in each batch were swabbed and reconstituted into pools. In summary, we have 18 pools of swabbed samples, 9 for the Novogen strain and 9 for the Lohman strain. For the treatment of the animals, we have considered EC council directives (EU Directive 2010/63/EU) regarding the protection of animals used for experimental and other scientific purposes. We also conducted all the experiments according to the protocol approved by the Ethical Committee of the Research Unit of Applied Microbiology and Pharmacology of Natural Substances.

### Bacteriological examination

After swabbing, the collected swabs were placed in Mueller Hinton broth for 4 h, followed by plating on Mueller Hinton agar. After 24 h of incubation, Gram staining was performed on each colony type present on the petri dish. The culture on MH agar medium followed by Gram staining allows us not to miss all the bacteria that would not have grown on selective media. According to the results of the Gram stain, eosin methylene blue (EMB) medium was plated for bacilli, and Chapman medium was plated for cocci. For *Salmonella*, gram-negative bacilli were streaked on xylose lysine deoxycholate (XLD) agar. The plates were incubated for 24 h at 37 °C [36].

### Bacterial identification

Colonies obtained on specific isolation media were subjected to Gram staining. Biochemical tests for identification included the oxidase test on gram-negative bacilli, followed by the Leminor gallery test when oxidase was negative. A confirmation of the results obtained with the Leminor Gallery was carried out with the API 20 E Gallery. Catalase, DNase and staphylocoagulase tests were performed on gram-positive cocci. Catalase-negative cocci were plated on fresh blood agar to characterize hemolysis [37].

### Antimicrobial resistance profile

Antibacterial susceptibility screening to antibiotics was conducted using the Kirby–Bauer disc diffusion method. The results were interpreted according to guidelines from the European Committee on Antimicrobial Susceptibility Testing (EUCAST) [38]. The antibiotic discs used for gram-negative bacilli were amoxicillin + clavulanic acid (AMC): 30 µg, amoxicillin (AMX): 25 µg, cefotaxime (CTX): 30 µg, aztreonam (AT): 30 µg, ciprofloxacin (CIP): 5 µg, gentamicin (GEN): 10 µg, ceftriazone (CRO): 30 µg, imipenem (IMP): 10 µg, ertapenem (ARE): 10 µg, colistin (CS): 50 µg, and trimethoprim + sulfamethoxazole (SXT): 25 µg. For staphylococci, we used oxacillin (OX): 5 µg, erythromycin (E): 15 µg, vancomycin (VA): 30 µg, vancomycin (VA): 30 µg, teicoplanin (TEC): 30 µg, tobramycin (TOB): 10 µg, amoxicillin (AMX): 25 µg, cefoxitin (FOX): 30 µg, and gentamicin (GEN): 10 µg. Antibiotic discs used for Enterococci are oxacillin (OX): 5 µg, vancomycin (VA): 30 µg, teicoplanin (TEC): 30 µg, and ceftriaxone (CRO): 30 µg.

### Real-time PCR to detect resistance genes

DNA extraction was performed by the boiling method [39]. Resistance genes were screened by real-time PCR. Each PCR consisted of 10 µl of Luna Universal qPCR master mix, 0.5 µl of each primer (10 µM), 6 µl of water and 3 µl of DNA template. Real-time PCR was performed using a LineGene9600 Plus fluorescent quantitative detection system (Hangzhou Bioer Technology, China) with the following program: 95 °C for 60 s, 40 cycles consisting of (i) 95 °C for 15 s and (ii) annealing temperature for 15 s, and a melting step consisting of (i) 95 °C for 15 s, (ii) melting temperature for 60 s and (iii) 95 °C for 15 s. Cycle thresholds (CTs) were reported, and positive samples were isolates with a cycle threshold below 30. Primer sequences are presented in Table 1.

**Table 1:** Primer sequences

Gene	Sequence of the primer	Authors
TEM	F : TCGGGGAAATGTGCG R : GGAATAAGGGCGACA	(De Gheldre et al. 2003)
SHV	F : AAGATCCACTATCGCCAGCAG R : ATTCAGTTCCGTTTCCCAGCGG	(Memariani et al. 2015)
CTXMI	F : GGTTAAAAAATCACTGCGTC R : TTGGTGACGATTTTAGCCGC	(Memariani et al. 2015)
GES	F : GCAATGTGCTCAACGTTCAAG R : GTGCCTGAGTCAATTCTTTCAAAG	(Ichola et al. 2021)
KPC	F: GCCGCCAATTTGTTGCTGAA R: GCCGGTCGTGTTTCCCTTT	(Ichola et al. 2021)
NDM	F: GGCCACACCAGTGACAATATCA R: CAGGCAGCCACCAAAAGC	(Ichola et al. 2021)
IMP	F: GGAATAGAGTGGCTTAATTC R: GCTTTAACAAAAACAACCACC	(Ichola et al. 2021)
VIM	F: GCACTTCTCGGCGGAGATTG R: CGACGGTGATGCGTACGTT	(Ichola et al. 2021)
OXA23	F: TTTACTTGCTATGTGGTTGCT R: ATCACCTGATTATGTCCTTGA	(Ichola et al. 2021)
OXA48	F: TGTTTTTGGTGGCATCGAT R: GTAAMRATGCTTGGTTCGC	(Ichola et al. 2021)
MCR1	F : CACATCGACGGCGTATTCTG R : CGATGTCGGTATGCTCGTTG	(Waseem 2019)
Sul I	F : CGCACCGGAAACATCGCTGCAC R : TGAAGTTCCGCCGCAAGGCTCG	(Pei et al. 2006)
Sul II	F: TCCGGTGGAGGCCGGTATCTGG R: TCCGGTGGAGGCCGGTATCTGG	(Pei et al. 2006)

### Data analysis

Data on poultry wise status about the presence of bacterial isolates and sensitivity to different antibiotics were entered into an Excel spreadsheet 2013. The prevalence of gram-negative bacilli, staphylococci and enterococci was summarized as frequencies and percentages. Similarly, susceptibility to various antibiotics was presented as frequencies and percentages. If the organism was resistant to three or more classes of antibiotics, it was considered to be multidrug resistant (MDR) [40].

## Data Availability

The data are available upon request with the corresponding author.

## Abbreviations

AMC: amoxicillin + clavulanic acid
AMX: Amoxicillin
ARE: Ertapenem
AT: aztreonam
CTX: Cefotaxime
CIP: Ciprofloxacin
CRO: Ceftriaxone
CS: Colistin
E: Erythromycin
EUCAST: European Committee on Antimicrobial Susceptibility Testing
FOX: Cefoxitin
GEN: gentamicin
IMP: imipenem
MH: Mueller Hinton
OX: Oxacillin
PCR: Polymerase chain reaction
SPSS: Statistical Package for the Social Sciences
TEC: Teicoplanin
TOB: Tobramycin
VA: Vancomycin
XLD: Xylose Lysine Decarboxylase
